# Analysis and Synthesis of Natural Texture Perception From Visual Evoked Potentials

**DOI:** 10.3389/fnins.2021.698940

**Published:** 2021-07-26

**Authors:** Taiki Orima, Isamu Motoyoshi

**Affiliations:** ^1^Department of Life Sciences, The University of Tokyo, Tokyo, Japan; ^2^Japan Society for the Promotion of Science, Tokyo, Japan

**Keywords:** image statistics, visual evoked potentials, texture perception, stimulus reconstruction, naturalness perception

## Abstract

The primate visual system analyzes statistical information in natural images and uses it for the immediate perception of scenes, objects, and surface materials. To investigate the dynamical encoding of image statistics in the human brain, we measured visual evoked potentials (VEPs) for 166 natural textures and their synthetic versions, and performed a reverse-correlation analysis of the VEPs and representative texture statistics of the image. The analysis revealed occipital VEP components strongly correlated with particular texture statistics. VEPs correlated with low-level statistics, such as subband SDs, emerged rapidly from 100 to 250 ms in a spatial frequency dependent manner. VEPs correlated with higher-order statistics, such as subband kurtosis and cross-band correlations, were observed at slightly later times. Moreover, these robust correlations enabled us to inversely estimate texture statistics from VEP signals via linear regression and to reconstruct texture images that appear similar to those synthesized with the original statistics. Additionally, we found significant differences in VEPs at 200–300 ms between some natural textures and their Portilla–Simoncelli (PS) synthesized versions, even though they shared almost identical texture statistics. This differential VEP was related to the perceptual “unnaturalness” of PS-synthesized textures. These results suggest that the visual cortex rapidly encodes image statistics hidden in natural textures specifically enough to predict the visual appearance of a texture, while it also represents high-level information beyond image statistics, and that electroencephalography can be used to decode these cortical signals.

## Introduction

The visual field is full of complex image regions called “textures.” Increasing evidence shows that textural information, or ensemble statistics, play a key role in the rapid perception and recognition of scenes, objects, and surface materials (Lowe, 1999; Oliva and Torralba, 2001; Motoyoshi et al., 2007; Whitney et al., 2014; De Cesarei et al., 2017; Fleming, 2017; Nishida, 2019).

It has widely been suggested that the perception of a texture is essentially based on the spatial distributions of low-level image features and their relationships (Julesz, 1965; Graham et al., 1992; Landy and Graham, 2004). Following extensive investigations into the neural computations underlying texture segregation (Bergen and Adelson, 1988; Zipser et al., 1996; Baker and Mareschal, 2001), recent studies have re-formalized the theory in terms of image statistics (Portilla and Simoncelli, 2000; Freeman and Simoncelli, 2011; Freeman et al., 2013; Wallis et al., 2017). Specifically, the early visual cortex decomposes an image into multiple subbands of different orientation and spatial frequency, encodes moment statistics and correlations across subbands of different orientation and spatial frequency, and exploits these statistics to discriminate among various texture images. Compelling evidence for this framework is provided by texture-synthesis algorithms (Heeger and Bergen, 1995; Portilla and Simoncelli, 2000), which can synthesize a texture image that looks similar to a given texture by simply matching image statistics of white noise to those of the target texture.

Recent studies adopting functional magnetic resonant imaging and electrophysiology suggest that texture statistics are represented in the early visual cortex (Freeman and Simoncelli, 2011; Freeman et al., 2013; Okazawa et al., 2015, 2017). Yet, it is unclear how each class of statistic is encoded in the human brain, especially during the early processing of the image. To examine such a rapid cortical response in humans, electroencephalography (EEG) has widely been used as an easy and non-invasive measure. In visual neuroscience, classical studies have examined visual evoked potentials (VEPs) for a specific image feature, but with artificial patterns composed of lines and dots (Victor and Conte, 1991; Bach and Meigen, 1997, 1998; Peterzell and Norcia, 1997; Bach et al., 2000; Norcia et al., 2015; Kohler et al., 2018). More recently, several studies directly measured VEPs for natural images. Adopting reverse correlation analysis (DeAngelis et al., 1993), they successfully extracted VEP components correlated to particular image features, such as pixel statistics, phase statistics, the scene “gist,” and deep features (Rousselet et al., 2008; Scholte et al., 2009; Bieniek et al., 2012; Groen et al., 2012a, b, 2017; Hansen et al., 2012; Ghodrati et al., 2016; Greene and Hansen, 2020). However, these features are not powerful enough to fully describe the perception of individual images of scenes and objects they employed, and it is uncertain if the VEP components correlated with those features are truly relevant to the perception. In addition, those features are indifferent to texture perception.

In contrast to the perception of scenes and objects, the perception of textures is well described and even synthesized by a particular set of image statistics (Portilla and Simoncelli, 2000). Moreover, such image statistics are spatially global measurements, whose neural representations could be captured by EEG with a low spatial resolution. Taking advantage of these facts, the present study elucidates human cortical responses to texture statistics using a reverse correlation between VEPs for various natural textures and image statistics that are critical for the perceptual appearance of a texture. Our analysis revealed VEP components specifically correlated with low- and high-level texture statistics. On the basis of this robust correlation, we reconstructed image statistics from VEPs with linear regression and successfully synthesized perceptually mimicked textures simply from VEP signals. These results suggest that VEPs can capture neural responses to texture statistics specifically enough for the prediction of the perceptual appearance of individual images. We found different VEPs between natural textures and their synthetic versions, but those VEPs were limited to images in which texture statistics were not sufficient to synthesize the appearance of natural textures.

## Materials and Methods

### Observers

Fifteen naïve, paid observers (22 years old on average) participated in the experiment. All participants had normal or corrected-to-normal vision. All experiments were conducted in accordance with the guidelines of the Ethics Committee for experiments on humans at the Graduate School of Arts and Sciences, The University of Tokyo. All participants provided written informed consent.

### Apparatus

Visual stimuli were displayed on a gamma-corrected 24-inch liquid-crystal display (BENQ XL2420T) with a frame rate of 60 Hz. The pixel resolution was 1.34 min/pixel at a viewing distance of 100 cm, and the mean luminance of the uniform background was 33 cd/m^2^.

### Stimuli

**FIGURE 1:**
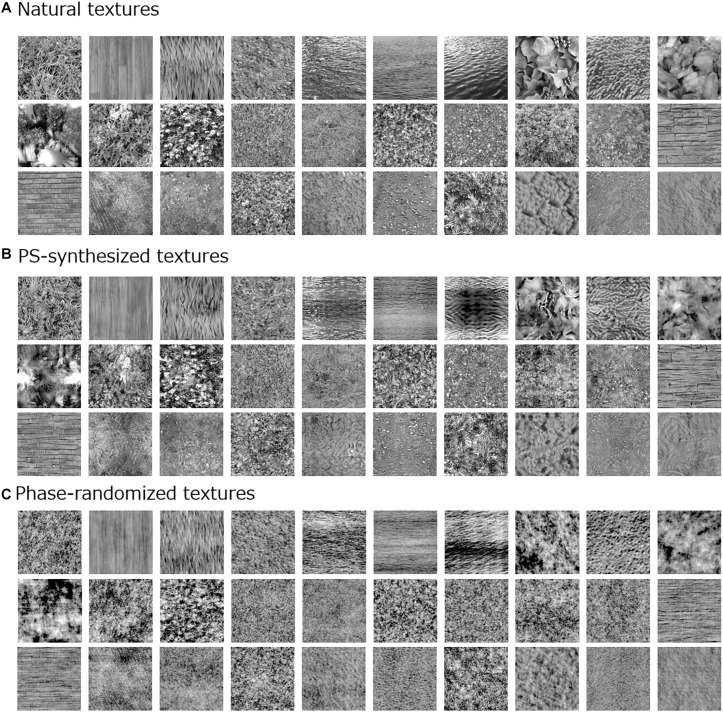
Examples of visual stimuli used in the experiment: **(A)** natural textures; **(B)** Portilla–Simoncelli (PS)-synthesized versions; and **(C)** phase-randomized versions.

The visual stimuli comprised 166 natural texture images, each subtending 5.7° × 5.7° (256 × 256 pixels; Figure 1A). Images were taken from our original natural-texture image database or from the Internet. All RGB images were converted to gray scale, and the mean luminance was normalized to 33 cd/m^2^, which was equal to that of the gray background.

For comparison with the original natural textures, we additionally employed two types of synthesized image. One was an image synthesized by means of the Portilla–Simoncelli (PS) algorithm (Figure 1B; Portilla and Simoncelli, 2000), which can create a perceptually similar texture by matching low- and high-level image statistics of a white noise image, including moment statistics [i.e., standard deviation (SD), skew, and kurtosis] and cross-band correlations, to those of the original texture image. The synthesis was performed with a typical parameter setting as used in the original algorithm (except for the number of iterations) (Portilla and Simoncelli, 2000). The other synthetic textures were made by randomizing the spatial phase of the original natural textures (Figure 1C). These phase-randomized images were equivalent to the original image only in terms of the global spatial frequency spectrum.

### Procedure

Electroencephalographys were measured in an electrically shielded, dark room. In each experimental session, each of 166 natural textures was presented once in random order, with a 500-ms duration followed by a 750-ms interval of the uniform gray background. Observers viewed the stimulus binocularly with steady fixation on a small black dot (10.8-min in diameter) that was shown at the center of the display throughout the session. For each observer, the sessions were repeated 24 times. The same measurements were also run as different blocks for the PS-synthesized textures and for the phase-randomized textures. Each block was conducted in the same order for all participants on different days. Therefore, each observer spent 3 days in total participating in the EEG recordings (i.e., measurements were made for natural textures on the first day, PS-synthesized textures on the second day, and phase-randomized textures on the third day).

### EEG Recordings and Preprocess

The EEG recordings were conducted using electrodes positioned at Fp1, Fp2, F3, F4, C3, C4, P3, P4, O1, O2, F7, F8, T7, T8, P7, P8, Fz, Cz, and Pz, in accordance with the international 10–20 system, at a 1,000-Hz sampling rate, using Ag-AgCl electrodes and an electrode cap of appropriate size (BrainVision Recorder, BrainAmp amplifier, EasyCap; Brain Products GmbH). An additional electrode, which served as the common ground electrode, was placed midway between Fz and Fpz. All electrodes were referenced to another electrode positioned between Fz and Cz, and they were re-referenced off-line using the average amplitude of all electrodes. The EEG was resampled at 250 Hz, band-pass filtered at 0.1–100 Hz, and converted to epochs of −0.4 to 0.8 s from the stimulus onset. The power frequency component (50 Hz) was automatically rejected when the EEG was recorded. The baseline was from −0.1 to 0 s with respect to the stimulus onset, and the EEG was corrected relative to the baseline. Artifact components (i.e., eye movements) were removed by the heuristic examination of independent components. To remove epochs with eye blinks, epochs with an amplitude outside the range from −75 to 75 μV (i.e., 1.7% of all epochs) were rejected. VEPs for each image were defined as the average across the 24 repetitions. We compensated for machinery delay that was measured in each trial.

### Analysis of Image Statistics

We analyzed image statistics for each texture image. In the analysis, the PS statistics space was not used directly because it was primarily designed for synthesis and consists of very complicated combinations of parameters, which are not suitable for visualizing the results. Instead, we chose several classes of statistics that are known to be particularly important in human texture models, including the PS model (Portilla and Simoncelli, 2000; Simoncelli and Olshausen, 2001; Landy and Graham, 2004). In any natural image, some of these statistics may be correlated with each other, but we defined them as independent classes in terms of their properties. Thus, we decomposed each image into different orientation and spatial frequency subbands and computed five representative image statistics: the SD, skew, kurtosis, correlation between different orientation subbands, and correlation between different spatial frequency subbands. In this space, we confirmed that natural textures and their PS-synthesized versions had almost identical, or very similar, image statistics (*r* = 0.83 on average).

**FIGURE 2:**
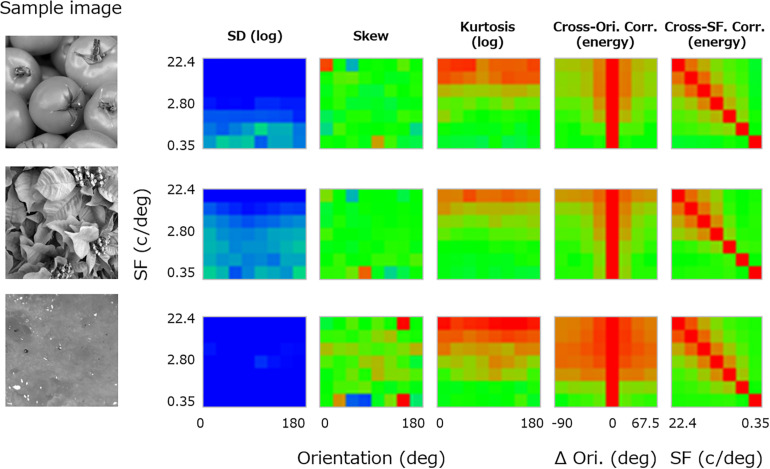
Image statistics calculated for a sample texture image (far left). From the left, the central three panels show the log SD, skewness, and log kurtosis plotted as functions of the spatial frequency and orientation of the subband. The two right-most panels show the cross-orientation energy correlation plotted as a function of the spatial frequency and the orientation (Ori) difference between subbands, and the cross-frequency energy correlation plotted as a function of the spatial frequency (SF) and the paired SF. The color of each pixel represents the value of the statistics, separately scaled for each class of statistics.

For each texture, the luminance image was first decomposed to subbands of seven spatial frequencies (2–128 cycles/image, 1-octave steps: 0.35, 0.70, 1.40, 2.80, 5.61, 11.2, and 22.4 cycles/deg) (e.g., De Valois and De Valois, 1980) and eight orientation bands (0–157.5°, 22.5° steps) by using a linear Gaussian band-pass filter with a spatial frequency bandwidth (i.e., full width at half-maximum) of 1 octave and an orientation bandwidth of 30°. For each subband image, three moment statistics (i.e., log SD, skewness, and log kurtosis) were calculated. The central three panels in Figure 2 show these three moment statistics obtained from a sample image (left-most image in Figure 2) and plotted as functions of orientation (*x*-axis) and spatial frequency (*y*-axis). We did not consider pixel statistics because visual cortical neurons have no direct access to pixel information.

In addition, correlations between subband “energy” images of different orientation and spatial frequency were calculated. These are known to be important high-level image statistics in texture synthesis (Portilla and Simoncelli, 2000). In detail, the cross-orientation energy correlations are related to how much local features in the image are oriented, and the cross-frequency energy correlations are related to how much the local luminance modulations are edgy or stepwise (Portilla and Simoncelli, 2000; Balas et al., 2009). Here, the energy image was given as a vector sum of the cosine and sine parts of the subband image. We calculated correlations in the energy image between different orientation bands along the same spatial frequency and between different spatial frequency bands along the same orientation. We then averaged the resulting correlations across orientation because the absolute orientation rarely matters in texture perception.

Specifically, we computed the “cross-orientation correlation” (*XO*) between subbands of variable orientation difference (Δθ) at each spatial frequency (f) according to Eq. 1. The panel second from the right in Figure 2 shows the resulting cross-orientation correlation plotted as a function of Δθ (*x*-axis) and f (*y*-axis).

(1) $$XO_{\Delta \theta ,f}=\sum_{\theta }\frac{corr\left(w_{\theta ,f},w_{\theta +\Delta \theta ,f}\right)}{K}$$

In a similar manner, we also computed the “cross-frequency correlation” (*XF*) for the difference of a variable pair of spatial frequencies (f and f′) according to Eq. 2. The right-most panel in Figure 2 shows the resulting cross-frequency correlation plotted as a function of f′ (*x*-axis) and f (*y*-axis).

(2) $$XF_{f,f^{\prime }}=\sum_{\theta }\frac{corr\left(w_{\theta ,f},w_{\theta ,f^{\prime }}\right)}{K}$$

Here, *K* is the number of orientations, corr stands for the correlation coefficient, and θ is the orientation of the subband.

We did not adopt correlation between “linear” subbands in our analysis because it had an extremely small variation across images (i.e., the variance was approximately 1/256 of that of energy subbands) owing to the narrow bandwidth of the spatial filters that we used, i.e., 30° in orientation and 1 octave in spatial frequency. While the linear cross-scale correlation is closely related to the cross-scale phase statistics and important in representing “edgy” structures in the image (Concetta Morrone and Burr, 1988; Kovesi, 2000; Portilla and Simoncelli, 2000), it plays a small role in texture perception unless one scrutinizes the image at the fovea (Balas, 2006; Balas et al., 2009).

### Partial-Least-Squares Regression Analysis

To obtain the regression model for the VEPs and the image statistics of the visual stimulus, we conducted a partial-least-squares regression analysis between them. We assigned the VEPs to the predicator and the image statistics to response variables. We implemented the SIMPLS algorithm through the MATLAB function “plsregress”. There were seven components, which minimized the prediction error of the response in a 10-fold cross validation in the training set (The mean squared error of the response was 80.0).

## Results

### VEPs

**FIGURE 3:**
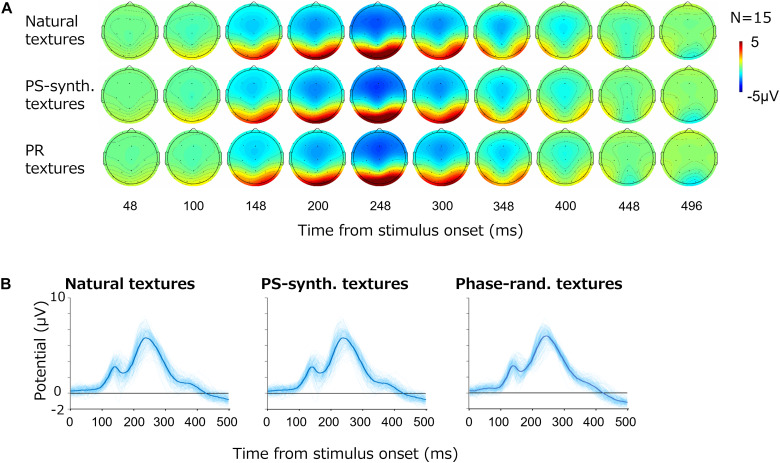
Visual evoked potentials (VEPs) for texture images. **(A)** Topography of the grand average VEPs for the natural textures, PS-synthesized textures, and phase-randomized (PR) textures, in rows from top to bottom. **(B)** VEPs at the occipital electrodes (mean of O1 and O2). The light-blue traces show VEPs for individual images and the thick blue traces represent the averages across images.

Figure 3A shows the average VEPs for all images. Each row shows the results for one image type; i.e., natural textures, PS-synthesized textures, and phase-randomized textures. For all types, large-amplitude VEPs (∼10 μV) were observed at the occipital electrodes (O1/O2). As we did not find any systematic and independent components in the other cortical regions, we here focus on VEPs from those two occipital electrodes.

Figure 3B shows the time course of VEP amplitudes at the occipital electrodes (i.e., the averaged responses from O1 and O2) for the different types of stimuli. The light-blue curves show the average VEPs for the individual images whereas the thick blue curves are the VEPs averaged across all images. The potentials at the occipital electrodes began to rise at 100 ms after the stimulus onset and reached a first small peak at around 120 ms followed by a second large peak at around 250 ms. The basic waveforms were also similar across images, but there were large variations across individual textures.

### Correlation Between VEPs and Image Statistics

We conducted a reverse-correlation analysis of the VEPs and each image statistic. We conducted the reverse-correlation analysis for individual observers but the resulting data were noisy and lacking in robustness. This was thought to be because the number of repetitions for each image (24 repetitions) was small for the reverse-correlation analysis. To address this problem, in accordance with the method used in the previous studies (Scholte et al., 2009; Hansen et al., 2011), we computed z-scored VEPs at each time point for each observer and averaged them across observers. We then computed the coefficient of correlation between each image statistic and the z-scored VEP at each time point.

**FIGURE 4:**
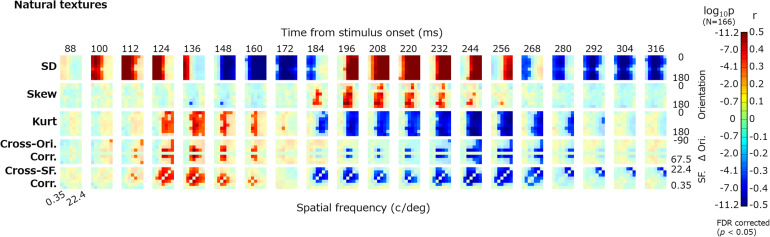
Correlations between image statistics and VEPs. The rows from top to bottom show the correlation of VEPs with the log SD, skew, log kurtosis, cross-orientation correlation, and cross-frequency correlation. Red indicates positive correlations and blue indicates negative correlations. The colors are desaturated for values that are not statistically significant (*p* ≥ 0.05, FDR corrected). The format of each panel follows that in Figure 2. The maps are arranged in columns for different time points, from 88 to 316 ms. SF, spatial frequency; Ori, orientation; and *r*, correlation coefficient.

Figure 4 shows the dynamics of the correlations between image statistics and occipital VEPs (O1/O2). Each pixel in the heatmap indicates the coefficient of correlation between the VEPs at a particular timepoint (e.g., 100 ms) and a particular image statistic (e.g., log SD at 0-deg orientation and 2-c/image spatial frequency). Red indicates a positive correlation and blue indicates a negative correlation. Progressing downward, each row shows the results for a class of image statistics; i.e., log SD, skew, log kurtosis, cross-orientation correlation, and cross-frequency correlation. To address the multiple comparisons among time points and image statistic parameters, we adopted the Benjamini–Yekutieli false discovery rate (FDR)-correction method (Benjamini and Yekutieli, 2001). The significant correlations (FDR-corrected, *p* < 0.05) are indicated by vivid colors.

For all classes of image statistics, we found strong correlations with the VEPs that systematically develop over time. For instance, the VEPs had a strong positive correlation with the low-spatial-frequency SDs from ∼100 to ∼150 ms, a negative correlation with the mid-/high-spatial-frequency SDs from ∼150 to ∼180 ms, and a positive correlation with the mid-/high-spatial-frequency SDs from ∼190 to ∼260 ms. Such systematic rises and falls of correlations were found for the other classes of image statistics, with different timing. As we had obtained maps of the correlation dynamics for VEPs from other electrodes (F3, Fz, F4, P7, and P8), we confirmed that they were all similar to, or just sign-reversed from, the results obtained for the occipital electrodes (Figure 4).

### Correlation Between VEPs and Summarized Image Statistics

The correlation maps shown in Figure 4 appear somehow redundant. Regarding the moment statistics, for instance, the correlations with VEPs are nearly constant across all absolute orientations, as expressed by vertical “bands” in the maps. For the cross-band correlations, the absolute correlation with VEPs was always higher where the target subbands were close together in orientation (i.e., small Δθ) and in spatial frequency (small | f-f′|), which is expressed as diagonal spreading on the maps. This is not surprising given that VEPs can hardly resolve a neural response across different absolute orientations. In addition, the absolute orientation plays a small role in the visual appearance of a texture. Accordingly, we calculated the correlations between the VEPs and further summarized measurements, so that we could interpret the temporal dynamics of VEPs correlated with each class of image statistics more easily. To that end, the summarized moment statistics (i.e., log SD, skew, and log kurtosis) were defined as the averages across the orientation for each spatial frequency. The summarized cross-orientation correlation was given as the average across-orientation difference (Δθ except Δθ = 0) for each spatial frequency. The summarized cross-frequency correlation was given as the average across-frequency difference (f-f′ except f = f′).

**FIGURE 5:**
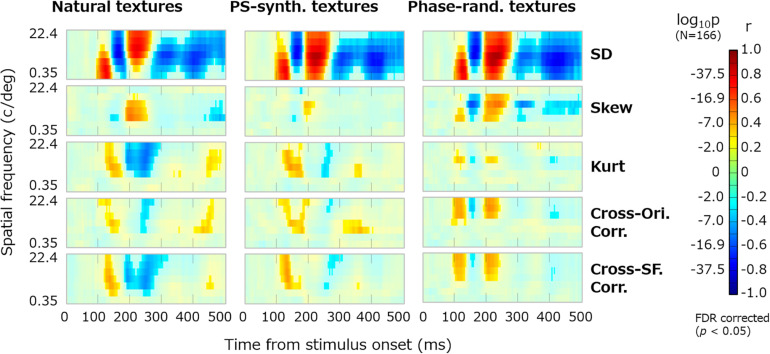
Dynamics of VEPs correlated with summary image statistics. The horizontal axes represent the time from the stimulus onset (0–496 ms) and the vertical axes represent the spatial frequency (c/deg). Reddish pixels indicate positive correlation and blueish pixels indicate negative correlation, for which non-significant data (*p* ≥ 0.05, FDR corrected) are desaturated. Panels in the successive rows show the correlations of VEPs with the SD, skew, kurtosis, and cross-orientation energy correlation and cross-frequency energy correlation. The results are shown for natural textures (left), PS-synthesized textures (middle), and phase-randomized textures (right).

Figure 5 shows the dynamics of correlation between VEPs and the summary image statistics. The results are shown for the three types of texture stimulus: natural, PS-synthesized, and phase-randomized textures. The vividly colored regions indicate statistically significant correlations identified using the Benjamini–Yekutieli FDR-correction method (*p* < 0.05). Similar patterns of the results were obtained for the other electrodes. We also confirmed that nearly the same results are obtained if we use image statistics calculated within the central or peripheral region in the image.

The temporal development of VEPs correlated with the summary image statistics is now clearly visible. VEPs correlated with SDs were particularly strong (*r*_*max*_ ≈ 0.8) and dynamically rose and fell in a spatial-frequency-dependent manner. They had a first peak at ∼120 ms for low-spatial-frequency bands (2–16 c/image), a second negative peak at ∼150 ms for middle spatial frequencies (4–64 c/image), and a third peak at ∼200 ms for high spatial frequencies (8–128 c/image). VEPs correlated to skewness were observed at ∼200 ms only for middle spatial frequencies (16–64 c/image). Even after 300 ms from the stimulus onset, we could observe significant correlations of VEPs to SDs and to some other statistics. VEPs correlated to kurtosis, cross-orientation correlation, and cross-frequency correlation appeared to have similar dynamics. They commonly tended to have a first positive peak at ∼150 ms and a second negative peak at ∼200–250 ms, but only for middle and high spatial frequencies. This similarity may be partly due to mutual correlations among the three statistics, which we confirmed not only for our texture stimuli but also for a wide range of natural images. However, as many texture models assume, they have independent roles in the perceptual discrimination of textures, and we confirmed that merging these VEP components prevented us from reconstructing textures from VEP signals.

The temporal dynamics of correlation were qualitatively similar across different types of image, that is, original, PS-synthesized, and phase-randomized images (Figure 5). The correlation maps in Figure 5 are highly correlated with each other; i.e., *r* = 0.83 (*p* ≈ 0) for the original and PS-synthesized textures. However, we still found a small difference in the results between the original and PS-synthesized textures despite the equality of image statistics between the two types of texture. We will discuss this difference later in detail.

### Reconstruction of Texture Image From the VEP

The series of analyses described above reveal a robust correlation structure between VEPs for natural textures and image statistics. This led us to the hypothesis that image statistics of a texture are predictable from VEP signals. In testing this possibility, we next sought to apply linear regression analysis, to inversely estimate the image statistics of texture stimuli from the VEP signals, and to determine if the estimated image statistics would enable us to synthesize images perceptually similar to the original texture. If such reconstruction was to be successful, it would further support the notion that the temporal pattern of VEPs for natural textures represents the neural processing of perceptually relevant image statistics.

For the purpose of texture synthesis from VEP signals, we adopted the texture statistics used in the PS texture-synthesis algorithm instead of the image statistics used in the above analyses (Note that most PS statistics are essentially equivalent or closely related to the image statistics used in the above reverse-correlation analysis). To construct a linear regression model of PS statistics and VEPs, we used partial-least-squares regression analysis. The number of statistics vectors in the PS texture space is too large to be used in such a regression model, and we therefore reduced PS statistics by applying a compression method inspired by a previous study (Okazawa et al., 2015): we set the number of orientation bands and number of scales each to 3, and the number of positions to 1; rejected the constant parameters; and utilized the symmetrical parameters in the cross-subband correlations. Thereafter, as mentioned in the section “Materials and Methods,” we chose to utilize these reduced PS-synthesis (cPS) parameters instead of the original PS statistics. We took VEPs for a period of 0–496 ms (125 points) as the predicator, and the cPS statistics (110 points) as the response variables. The training data set consisted of 299 natural and PS-synthesized texture images used in the experiment (about 90% of all the data), and the test set consisted of the remaining 33 texture images (about 10%). The regression model from the VEPs to the cPS-synthesis parameters was trained on the training set. There were seven components, which minimized the prediction error of the response in a 10-fold cross validation on the training set. Finally, the cPS statistics for the test set were predicted using the trained regression model.

The results indicate that cPS statistics were well predicted by the temporal pattern of VEP signals, suggesting a robust relationship between image statistics and VEPs, as also demonstrated by the reverse-correlation analyses above. *R*^2^ (train) was 0.35 and *R*^2^ (test) was 0.20. The correlation between reconstructed cPS statistics and original cPS statistics was 0.88.

**FIGURE 6:**
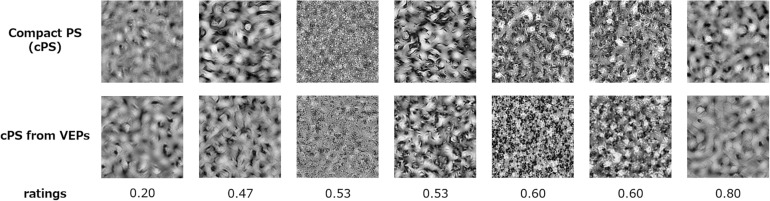
Compact Portilla–Simoncelli (cPS) synthesized textures and compact-PS-synthesized textures with the image statistics as estimated from VEPs. The perceptual dissimilarity ratings (0–4) are given below the images.

We synthesized textures using the estimated cPS statistics, to perceptually verify the quality of reconstruction. The synthesized textures are shown in Figure 6. The images in the upper row show the textures synthesized from the original cPS statistics, and the images in the lower row show textures synthesized with the cPS statistics as estimated from the VEPs. It is found that the VEP-based textures are very similar to, or almost indistinguishable from, the original cPS textures.

To obtain behavioral measures of this perceptual similarity between the original and VEP-based cPS textures, we had five observers (all of whom participated the EEG experiment) rate the quality of the VEP-based cPS textures in a separate experimental block after the EEG recordings. In the experiment, the original cPS textures and VEP-based cPS textures (7.8° × 7.8°) were displayed randomly on the left or right side on a uniform gray background of 40 cd/m^2^. The observers inspected the two textures with free viewing and rated their dissimilarity on a five-point scale; that is, from 4 (not similar at all) to 3 (not similar), 2 (similar), 1 (very similar), and 0 (hard to see the difference). For each observer, the rating was done with three repetitions for each of 31 of the 33 textures from the test dataset (The PS-synthesis algorithm did not work for two images). The results showed that the average dissimilarity rating across images was 2.04 (s.e. of 0.22), with an average cross-observer correlation of 0.90. Defining a rating of less than 2.0 as a successful synthesis, 52% of the textures were successfully synthesized from VEPs.

### Difference Between Natural and Synthetic Textures

**FIGURE 7:**
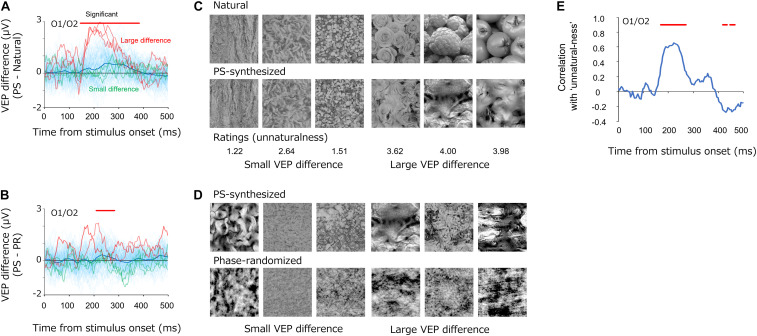
**(A,B)** Differential VEPs at the occipital electrodes (O1/O2) between the PS-synthesized textures and natural textures **(A)** and between the PS-synthesized textures and phase-randomized textures **(B)**. The light-blue traces are the differential VEPs for each texture. The red traces show the three largest differential VEPs and the green traces show the three smallest differential VEPs. **(C,D)** Pairs of textures that elicited small (left three images) and large (right three images) differential VEPs. Numbers below the images in **(C)** represent the average “unnaturalness” rating of the PS-synthesized texture. **(E)** Correlations between the differential VEPs and the perceptual unnaturalness ratings. The red bars indicate the statistically significant periods (*p* < 0.05, FDR-corrected).

While we observed that the average VEPs were similar among natural, PS-synthesized, and phase-randomized textures (Figure 3), we still found differences between the conditions with regard to individual images. Figure 7 shows the differential VEPs between natural and PS-synthesized textures (Figure 7A) and those between PS-synthesized and phase-randomized textures (Figure 7B). By means of the statistical test introduced by VanRullen and Thorpe Vanrullen and Thorpe (2001)(i.e., significant if *p* < 0.01 for 15 consecutive periods), we found a significant mean difference between the natural textures and PS-synthesized textures at 148–384 ms and between the PS-synthesized textures and phase-randomized textures at 212–284 ms. Meanwhile, we found a large variation in the differential VEPs across individual images (light-blue traces); i.e., large differential VEPs were found for some images but little or no difference for other images.

What gave rise to these variations in the differential VEPs? Whereas the PS synthesis successfully equalized image statistics in the natural textures for all images, it did not always successfully replicate the appearance of the natural texture and occasionally produced texture images that appeared unnaturalistic. Figure 7C shows example textures that produced small (left) and large (right) differential VEPs, on average, from 148 to 248 ms. Especially for the difference between natural and PS-synthesized textures, these pairs of images illustrate that synthesized textures that produced large differential VEPs appeared to be unnatural and perceptually unlike the original natural texture. These observations led us to the notion that variations in the differential VEPs are related to variation in the “unnaturalness” of PS-synthesized textures.

In testing this possibility, we carried out a simple rating experiment to measure the unnaturalness of each PS-synthesized texture in a separate experimental block after the EEG recordings. In that experimental block, all observers who participated in the EEG experiment used a five-point scale to rate how closely each PS-synthesized texture appeared like a photograph of a natural texture (0, almost the same as a natural texture; 1, similar to a natural texture; 2, a little dissimilar to a natural texture; 3, a little unnatural; 4, obviously unnatural). We also asked the observers to rate the unnaturalness of phase-randomized textures, but we found extremely high ratings (unnatural) for almost all images, and we therefore did not use those data in the analysis. The other experimental settings were the same as in the rating experiment for EEG-based texture synthesis.

We then analyzed how the perceptual unnaturalness of a synthesized texture was related to the differential VEP between the natural and PS-synthesized textures. Figure 7E shows the dynamics of correlation between the PS-synthesized minus natural differential VEPs and the unnaturalness ratings. Significant correlations (*p* < 0.05, FDR-corrected) were observed at a temporal epoch (168–268 ms) similar to that for the differential VEPs shown in Figure 7A. This indicates that PS-synthesized textures that looked unnatural gave rise to VEPs different from those of the original texture, even if they had nearly equal image statistics.

## Discussion

The present study investigated the temporal dynamics of cortical responses to biologically plausible image statistics of natural textures, by applying a reverse-correlation analysis between VEPs and image statistics. The analysis revealed that VEPs at the occipital electrodes are systematically correlated with image statistics that are known to be important for human texture perception. Moreover, on the basis of the robust relationship between the VEPs and image statistics, we successfully synthesized textures using image statistics as estimated from VEPs via a linear regression. These results support the notion that the human visual cortex rapidly encodes image statistics that play critical roles in the perception of natural textures. Although small differences were found for images that were not successfully synthesized, similar VEPs and correlation dynamics were observed for synthesized textures that had image statistics equivalent to those of the original natural textures.

Visual evoked potentials that correlated with the subband SD appeared in a spatial-frequency-dependent manner. They first peaked for low spatial frequencies at ∼100 ms after the stimulus onset, then peaked for middle spatial frequencies at ∼150 ms, and finally peaked for high spatial frequencies at ∼200 ms (Figure 5). This dynamic shift is consistent with “coarse-to-fine” processing, as suggested by a number of psychophysical studies on object/stereo processing (Schyns and Oliva, 1994; Hegdé, 2008). It is also consistent with physiological findings that magnocellular cells, which are tuned to low spatial frequencies, respond faster than parvocellular cells, which are tuned to high spatial frequencies (e.g., Nowak et al., 1995), and that the spatial frequency tuning of V1 cells shifts in a time-dependent manner from low to high spatial frequencies (Bredfeldt and Ringach, 2002; Mazer et al., 2002).

Visual evoked potentials also correlated with higher-order statistics, such as kurtosis and cross-subband energy correlations, with a similar temporal profile beginning as early as ∼120 ms after the stimulus onset. Considering the nature of each statistic, and past electrophysiological and psychophysical findings regarding texture processing, we speculate that these types of image statistic have a common functional and physiological basis. Kurtosis is primarily associated with spatial sparseness in the energy (complex-cell) outputs of a subband image (Kingdom et al., 2001; Olshausen and Field, 2004). As mentioned earlier, the cross-orientation energy correlations are related to the orientation of local features whereas the cross-frequency energy correlations are related to local luminance modulations (Portilla and Simoncelli, 2000; Balas et al., 2009). Neural computations for each of these three types of measurement are essentially based on inhibitory interactions among cortical neurons across space, orientation, and spatial frequency, respectively (Morrone et al., 1982; Ohzawa et al., 1982; Ferster, 1988; Zipser et al., 1996; Ferster and Miller, 2000; Nishimoto et al., 2006). These interactions are also functionally approximated as the second-order filters proposed in the human texture-vision model; i.e., filters that detect gradients of the energy output of a subband across space, orientation, and spatial frequency (Bergen and Adelson, 1988; Motoyoshi and Kingdom, 2003; Landy and Graham, 2004). It is likely that VEPs correlated with the three image statistics indicate the temporal dynamics of such interactive computations among neural channels in V1 and V2. It is not surprising that VEPs for such higher-order image statistics are observed at latencies as short as or only a little longer than those for SDs (except for very low spatial frequencies), given that the sharp orientation and spatial-frequency tuning of V1 cells emerges from the cross-channel interactions (Morrone et al., 1982; Ohzawa et al., 1982; Ferster and Miller, 2000).

The robust correlational structure between VEPs and image statistics allowed us to reconstruct texture images from image statistics that were inversely estimated from VEPs (Figure 6). In the present study, we deliberately applied a linear regression model even though it had lower prediction accuracy, in general, compared with prevailing non-linear “black box” models, including the deep neural network (DNN). Yet, the model we used still had an ability to reconstruct image statistics from occipital VEP signals accurately enough to synthesize textures that were perceptually similar to the target images. These results support the idea that the perceptual appearance of texture is ruled by such image statistics as encoded in the early visual cortex, and that the analysis of simple VEPs can extract these types of information.

While similar results were obtained for the natural and PS-synthesized textures, a small difference in VEP was found for some textures that were less successfully PS-synthesized and appeared “unnatural,” even though they had virtually equivalent image statistics (Figure 7). When we reanalyzed the dynamic correlations without such mal-synthesized stimuli (“unnaturalness” rating exceeding 3.0), at 88–300 ms after the stimulus onset, the results of the natural images and the PS-synthesized image were closer (with a root-mean-square error of 0.12) than those for the whole visual stimuli (with a root-mean-square error of 0.17). This result further supports the notion that VEPs largely reflect cortical responses to image statistics. However, it is noted that differential VEPs of unnatural textures were clearly observed for the period of 180–250 ms from the stimulus onset. This VEP component indicates that there is a rapid neural processing of information beyond image statistics. We also found significant differences in VEPs between PS-synthesized and phase-randomized textures. According to previous imaging (Freeman et al., 2013) and electrophysiological (Ziemba et al., 2019) studies, these differences could be related to differential neural processing in V1 and V2 for naturalistic textures.

The present study was limited to achromatic natural textures, and the texture image reconstruction was restricted to the texture perception that can be described by image statistics. Despite these limitations, the results of the present study demonstrated that reverse-correlation analysis, which focuses on the holistic features within a relatively large space, enabled us to extract the characteristics of the response of the visual cortex to natural “textures,” even with the low spatial resolution of EEG. In principle, the method proposed in the present study is general enough to be applicable to a wide variety of visual stimulus (e.g., natural scenes, materials, and objects) and image features [e.g., the spatial envelope, bags of features (such as the scale-invariant feature transform), and DNN features]. Future studies may extend the approach to better reconstruct the “’impression,” using non-linear models such as the DNN. The present study revealed that the impression of natural images is, at least partially, processed in the early visual cortex as statistical features. Therefore, according to the findings of the present study, the impression of a visual stimulus may be summarized as compact features, which would be beneficial in forming the basis for the efficient communication and display of real-world, complex natural images.

## Data Availability Statement

The raw data supporting the conclusions of this article will be made available by the authors, without undue reservation.

## Ethics Statement

The studies involving human participants were reviewed and approved by the Ethics Committee for experiments on humans at the Graduate School of Arts and Sciences, The University of Tokyo. The patients/participants provided their written informed consent to participate in this study.

## Author Contributions

TO and IM designed the research and wrote the manuscript. TO conducted the experiment and analyzed the data. Both authors contributed to the article and approved the submitted version.

## Conflict of Interest

The authors declare that the research was conducted in the absence of any commercial or financial relationships that could be construed as a potential conflict of interest.

## Publisher’s Note

All claims expressed in this article are solely those of the authors and do not necessarily represent those of their affiliated organizations, or those of the publisher, the editors and the reviewers. Any product that may be evaluated in this article, or claim that may be made by its manufacturer, is not guaranteed or endorsed by the publisher.
